# 8.K. Workshop: Pillars of health promotion and the role of Health literacy in the life course approach

**DOI:** 10.1093/eurpub/ckac129.511

**Published:** 2022-10-25

**Authors:** Organised by: EUPHA-HP, University of Education (Germany), Polytechnic Institute of Coimbra (Portugal), CrAdLiSa, Chair persons: Luís Saboga-Nunes (EUPHA-HP), João Lima (Portugal)

## Abstract

Three pillars are considered today for the development of Health Promotion: Good Governance, Healthy Cities and Health Literacy (HL) (The Shanghai Declaration, WHO 2016). Good Governance and policies to make healthy choices accessible and affordable to all, will ensure that all have the right to health. Sustainable environments are critical to make whole of society collaboration real and it is relevant that Cities become healthier settings. For this, increasing knowledge and social skills to help people make healthiest choices and decisions, i.e. Health Literacy (HL), emerges as the third pillar of the health promotion agenda today. The 10th WHO World conference in 2021 emphasized the role of HL in Health Governance (Stewarding for a flourishing future) that “... builds on co-design and makes full use of the digital transformation to achieve equitable benefits across populations, ensuring access and meaningful participation. This includes a high priority assigned to health literacy along the life course’ (WHO 2021). This workshop discusses how this third pillar (i.e. HL) may be translated into the everyday practice of Health Promotion “ensuring that people can cope with the challenges they face, through health literacy, ... to lead lives ... in harmony with Nature” (WHO 2021). From the public health perspective the aim of this workshop is to share with the audience an opportunity to immerse in four different social contexts and explore their HL practices as tracer approaches. The first context is set in Brazil and the emphasis is on HL role in increasing health knowledge. The authors share the results of a large research and an ethnographic analysis of the role of HL in the development of health decisions in the context of public libraries. The second presentation will unveil one of the topics that is influencing health and well-being today: the struggle to counter misleading information. Therefore, a study case from Portugal will focus on HL impact of nutritional marketing on the preferences, attitudes and consumption of children from 6 to 10 years old. A third presentation will consider food sustainability & health, and how HL may be expanded using simple and integrative pedagogical strategies in usual kindergarten dynamics to promote legumeś consumption. Understand how children construct their HL and how influenced they are in this process is the aim of the 4th presentation, where an analysis will be proposed in the quest of exploring HL on those in the first years of schooling. This workshop offers a forum for researchers, practitioners and policy-makers interested in health promotion pillars (e.g. like health literacy). By dialogue and two-way communication lively interaction and vivid discussions will be facilitated. This will allow discussing results regarding their benefit for improving policy research, practice, and policy-making, support further synergies, facilitate networking and collaboration, and support international capacity building.

**Key messages:**

• Refinement of concepts and practices (e.g. on health literacy) from south-north cultural contexts is needed for wider health promotion implementation strategies.

• The third pillar of Health Promotion (i.e. health literacy) demands a wider reflection of theoretical integrative approaches of health paradigms like salutogenesis.

